# Joint modeling of multivariate longitudinal data and survival data in several observational studies of Huntington’s disease

**DOI:** 10.1186/s12874-018-0592-9

**Published:** 2018-11-16

**Authors:** Jeffrey D. Long, James A. Mills

**Affiliations:** 1 Department of Psychiatry, Carver College of Medicine, University of Iowa, 500 Newton Road, Iowa City, IA 52242-1000 USA; 2 Department of Biostatistics, Department of Public Health, University of Iowa, 145 N. Riverside Drive, Iowa City, IA 52242-1000 USA

**Keywords:** Joint modeling (JM) - survival analysis - linear mixed modeling (LMM) - external validation - proportional hazards model - Huntington’s disease (HD)

## Abstract

**Background:**

Joint modeling is appropriate when one wants to predict the time to an event with covariates that are measured longitudinally and are related to the event. An underlying random effects structure links the survival and longitudinal submodels and allows for individual-specific predictions. Multiple time-varying and time-invariant covariates can be included to potentially increase prediction accuracy. The goal of this study was to estimate a multivariate joint model on several longitudinal observational studies of Huntington’s disease, examine external validity performance, and compute individual-specific predictions for characterizing disease progression. Emphasis was on the survival submodel for predicting the hazard of motor diagnosis.

**Methods:**

Data from four observational studies was analyzed: Enroll-HD, PREDICT-HD, REGISTRY, and Track-HD. A Bayesian approach to estimation was adopted, and external validation was performed using a time-varying AUC measure. Individual-specific cumulative hazard predictions were computed based on a simulation approach. The cumulative hazard was used for computing predicted age of motor onset and also for a deviance residual indicating the discrepancy between observed diagnosis status and model-based status.

**Results:**

The joint model trained in a single study had very good performance in discriminating among diagnosed and pre-diagnosed participants in the remaining test studies, with the 5-year mean AUC = .83 (range .77–.90), and the 10-year mean AUC = .86 (range .82–.92). Graphical analysis of the predicted age of motor diagnosis showed an expected strong relationship with the trinucleotide expansion that causes Huntington’s disease. Graphical analysis of the deviance-type residual revealed there were individuals who converted to a diagnosis despite having relatively low model-based risk, others who had not yet converted despite having relatively high risk, and the majority falling between the two extremes.

**Conclusions:**

Joint modeling is an improvement over traditional survival modeling because it considers all the longitudinal observations of covariates that are predictive of an event. Predictions from joint models can have greater accuracy because they are tailored to account for individual variability. These predictions can provide relatively accurate characterizations of individual disease progression, which might be important in the timing of interventions, determining the qualification for appropriate clinical trials, and general genotypic analysis.

## Background

Survival analysis is used to predict the timing of an event of interest, such as the death of a patient or the onset of dementia. Survival methods can handle the common situation of right-censoring, which arises when an individual experiences the event after the window of observation due to dropout or study termination (other types of censoring are possible). The usual endpoint or outcome of survival data consists of the two random variables of the time to the event and a censoring code (0 if censored, 1 if event).

Research questions in survival analysis often involve examining the extent to which covariates are valuable in prediction. If the covariates are repeatedly measured over time then it is most informative to use all the longitudinal data for prediction. Traditional survival analysis can be extended to incorporate time-dependent covariates [1], as long as the covariates have certain qualities. The covariates are required to be external, meaning their future values are known in advance and unaffected by the occurrence (or non-occurrence) of the event under study [2]. In addition, the covariates are assumed to be measured without error and measured at every event time to be analyzed [3]. Calendar date is an example of an external variable, as it can possibly be measured without error and measured at every event time. Once the date is assessed at study entry, it elapses in a perfectly predictable fashion and it is unaltered by an event.

In many longitudinal datasets, the covariates appear to be internal, reflecting changes of the intrinsic state of the patients under study. Future values of internal covariates are not pre-determined and their change is often related to the event. An example is disease signs and symptoms, which might increase over time to the extent that a diagnosis event occurs. Moreover, it seems regular for interval covariates to be measured with error and to be measured intermittently (not necessarily at every event time).

In order to properly incorporate internal covariates in prediction, it is desirable to use a joint model (JM) for the simultaneous analysis of the survival data of the event and the longitudinal data of the covariates. The foundations of JM can be approached from different perspectives, such as a special case of latent class analysis [4]. Another approach is the shared parameter framework [5], in which a survival submodel and a longitudinal submodel are interdependent through a set of shared random effects. Random effects are individual-specific model terms, and their inclusion in the JM provides a means of producing tailored predictions [6]. The tailored predictions are consistent with the concept of precision medicine [7] that considers individual variability in the predictions for a specific patient. The individual-specific information can be important for describing disease course, designing interventions, and phenotyping for subsequent genetic analysis.

The JM survival submodel can take a number of forms, with the proportional hazards model being a common choice. In this scenario, the goal of JM is to predict the hazard (or the log hazard) of the event in question. Covariates can be time-varying (i.e., longitudinal) or time-invariant. A time-invariant covariate is directly specified in the survival submodel similar to the traditional model. Each time-invariant covariate has a corresponding regression coefficient that indicates the strength of the covariate in predicting the hazard, adjusting for the other covariates. Rather than include a time-varying covariate directly in the survival submodel, the longitudinal information is specified through a function of a separate but interdependent longitudinal submodel for the covariate. A popular choice of function is the underlying or “true” value of the covariate that occurs contemporaneously with the hazard. Each true value predictor has a regression coefficient indicating its effect on the hazard.

The JM longitudinal submodel is a linear mixed model (LMM) for a continuous covariate, or a generalized LMM for a discrete covariate (e.g., binary variable). Our focus will be on the LMM, for which the aforementioned underlying value of the covariate is the linear predictor from the LMM that omits the random error term. That is, the linear predictor is a composite of both fixed and random effects, but not measurement error. Note that a time-varying covariate is the outcome in the LMM, but the true value of the covariate is a predictor in the JM survival submodel. It is in this manner that the two submodels are linked, with random effects being shared among the submodels.

When there is more than one time-varying covariate, then the LMM is said to be multivariate. Multivariate refers to multiple interdependent LMMs with different outcomes (the predictors in each LMM might or might not be the same). The different LMMs are interdependent through a shared random effects structure. In the JM survival submodel each true covariate is a predictor and has an associated regression coefficient indicating its strength of relationship with the hazard, adjusting for the other predictors in the model (whether they be other true covariate values or time-invariant covariates).

A fruitful area of application for JM is Huntington’s disease (HD). Several HD observational studies provide both survival endpoints as well as longitudinal internal covariates. HD is an inherited progressive neurodegenerative disease characterized primarily by motor disturbances, such as chorea. There is high interest in predicting the age of motor diagnosis and identifying covariates that are important in this prediction. The recent availability of data from several studies allows for external validation that can boost confidence that a particular JM might be applicable to a new HD sample. Tailored predictions from such validated models can be useful for characterizing the disease course of an individual and providing personalized phenotyping for subsequent genetic analysis.

In what follows, several practical issues of JM are discussed with HD as the area of application. We first consider model setup and estimation with two longitudinal covariates. Then external validation is discussed with focus on discrimination among those who are diagnosed and those who are not. Finally, we discuss individual-specific prediction and present types of predicted scores that are useful for HD research.

## Methods

### Huntington’s disease background and datasets

HD is caused by a mutation expansion of the cytosine-adenine-guanine (CAG) trinucleotide in the *HTT* gene of chromosome 2. Full penetrance occurs with CAG ≥ 40, but there is risk of developing HD with expansions as short as 36 [8]. HD is inherited in an autosomal dominant manner, meaning the mutation can be transmitted when at least one parent is a gene mutation carrier. The disease is characterized primarily by motor disturbances (e.g., chorea, rigidity), but cognitive deterioration (to eventual dementia) and behavioral disturbances (e.g., aggression) also occur. A landmark event in HD progression is motor diagnosis, which constitutes a very high confidence that the individual has sufficient manifest motor signs that can be fully attributed to the progression of HD. Motor diagnosis is determined by a trained rater (e.g., a neurologist) after a standard motor examination. There is a reliable genetic test for the CAG mutation expansion, which means that gene mutation expansion carriers can be identified pre-diagnosis, meaning prior to the manifestation of HD signs.

Four HD datasets were used in the analysis. Neuro-biological Predictors of Huntington’s Disease (PREDICT-HD) [9–13] is a longitudinal observational study of pre-diagnosis HD with 32 sites in six countries (AUS, CAN, DEU, ESP, GBR, USA). Data for the analysis was collected 2002–2014. Track-HD [14–16] is a longitudinal prospective observational study of pre-diagnosis and early HD with four sites in four countries (CAN, FRA, GBR, NE) with data collected 2008–2011. Enroll-HD [17] is a longitudinal observational study with participants who are pre-diagnosis, diagnosed with HD, and non-HD relatives and community controls. Data for the analysis was collected 2012–2016 from 61 sites in North America, Latin America, Europe, and Australasia. REGISTRY [18, 19] is a longitudinal observational study that includes pre-diagnosis participants, diagnosed participants, and at-risk individuals, with 89 European sites and data collected 2004–2012.

The analysis included individuals with a lab-verified HD gene mutation expansion who did not have a motor diagnosis at study entry. Additional inclusion criteria were ≥18 years of age (REGISTRY did not exclude Juvenile HD), a CAG ≥40 (CAG range among studies was 36–66), and complete data on the variables for the analysis. Sample sizes and descriptive statistics for key variables at study entry are shown in Table 1.

**Table 1:** Descriptive statistics for variables measured at study entry. Mean (SD) for quantitative variables and proportion for categorical variables

	Enroll-HD	PREDICT-HD	REGISTRY	Track-HD
*N*	643	873	481	150
*N*_obs_	1.44(0.67)	4.16(2.73)	2.09(1.43)	3.80(1.85)
Age at Entry	40.23(11.40)	39.69(9.75)	40.60(11.14)	40.57(8.34)
Age at Event	41.77(11.38)	44.64(10.28)	43.61(11.01)	44.47(8.57)
Diagnosis	0.16	0.26	0.40	0.33
Female	0.61	0.64	0.56	0.54
CAG	42.54(1.94)	42.51(1.98)	42.76(2.00)	42.99(1.94)
TMS	4.37(5.75)	4.83(5.13)	4.72(6.89)	2.89(2.16)
SDMT	48.86(13.01)	51.02(11.69)	45.11(13.82)	51.93(9.95)

Note. *N*_obs_ number of observations per participant, *CAG* cytosine-adenine-guanine expansion, *TMS* total motor score, *SDMT* symbol digit modalities test

All the studies had annual visits. At each visit a motor exam was conducted, the standard HD assessment battery was administered (Unified Huntington’s Disease Rating Scale [20]), and a determination of motor diagnosis was made (coded as 0 = no diagnosis, 1 = diagnosis).

Enroll-HD is an on-going and open-ended study. Some participants from the other studies were known to transition to Enroll-HD. In order to make the studies as independent as possible, the data from participants known to transition were omitted from Enroll-HD. That is, only data from the participants' originating study were used in the analysis.

### Joint model setup

The JM considered here is an extension of a traditional proportional hazards model previously used in HD research [21]. For purposes of simplicity, HD survival models often use the time metric of years from study entry to motor diagnosis, with covariates measured only at entry [22]. In one such model [21], the hazard of motor diagnosis (instantaneous rate of motor diagnosis) was a function of an unspecified baseline hazard, a CAG and age at study entry expression (see below), total motor score (TMS), and the symbol digit modalities test (SDMT). The TMS and SDMT are clinical variables collected as part of the standard HD battery. The TMS indexes the extent of motor impairment (e.g., chorea, bradykinesia) based on the standard motor exam, and it ranges from 0 (normal) to 124 (severest impairment). The SDMT is a timed symbol-matching task that measures working memory, complex scanning, and processing speed, with higher scores indicating better performance, and 0 indicating the worst possible performance.

To set up the proportional hazards model, we represent survival data in the following manner. Let ${T}_i^{\ast }$ be the true time of motor diagnosis for the *i*^*th*^ patient (*i* = 1, …, *N*), and let *C*_*i*_ be the right-censoring time. The observed time is ${T}_i=\mathit{\min}\left({T}_i^{\ast },{C}_i\right)$, and the censoring indicator is ${\delta}_i=I\left({T}_i^{\ast}\le {C}_i\right)$, which takes the value of 1 for motor diagnosis and 0 for censoring.

The proportional hazards model from previous research is

1 $$ {h}_i\left({t}^{\star}\right)={h}_0\left({t}^{\star}\right)\mathit{\exp}\left\{{\gamma}_1{\mathtt{CAP}}_i+{\gamma}_2{\mathtt{TMS}}_i+{\gamma}_3{\mathtt{SDMT}}_i\right\},\kern3.00em $$

where *t*^⋆^ is time on study in years, with *t*^⋆^ = 0 being study entry (the time origin); *h*_0_(*t*^⋆^) is the unspecified baseline hazard, with “baseline” here referring to the situation in which all the covariates are 0; and CAP is the CAG-Age Product (CAP), a well-established progression index in HD [23], defined as ${\mathtt{CAP}}_i={\mathtt{AGE}}_i\left({\mathtt{CAG}}_i-33.66\right)$, with age measured only at study entry. CAP was included rather than separate terms for CAG and age in order to be consistent with previous research [13, 21].

In a past analysis, the proportional hazards model of Eq. 1 was validated on multiple HD datasets, and it showed a relatively strong ability to discriminate among patients with different times of diagnosis [21]. Though discrimination has some drawbacks, such as not using all the data when there is censoring [24], the ability to correctly distinguish among patients with different timings of diagnosis is highly desirable in HD research [25].

The model of Eq. 1 has years on study as the time metric. A goal of the current analysis is to predict the age at motor diagnosis, with age being the natural time metric for this purpose. A second issue regarding Eq. 1 is that only the covariate values at study entry are considered. Because the TMS and SDMT are well-known indexes of disease progression, it is desirable to use the complete longitudinal record of these covariates in prediction. The covariates are internal because their change is related to a higher probability of motor diagnosis, which is a condition that is favorable for the use of a JM.

**Fig. 1:**
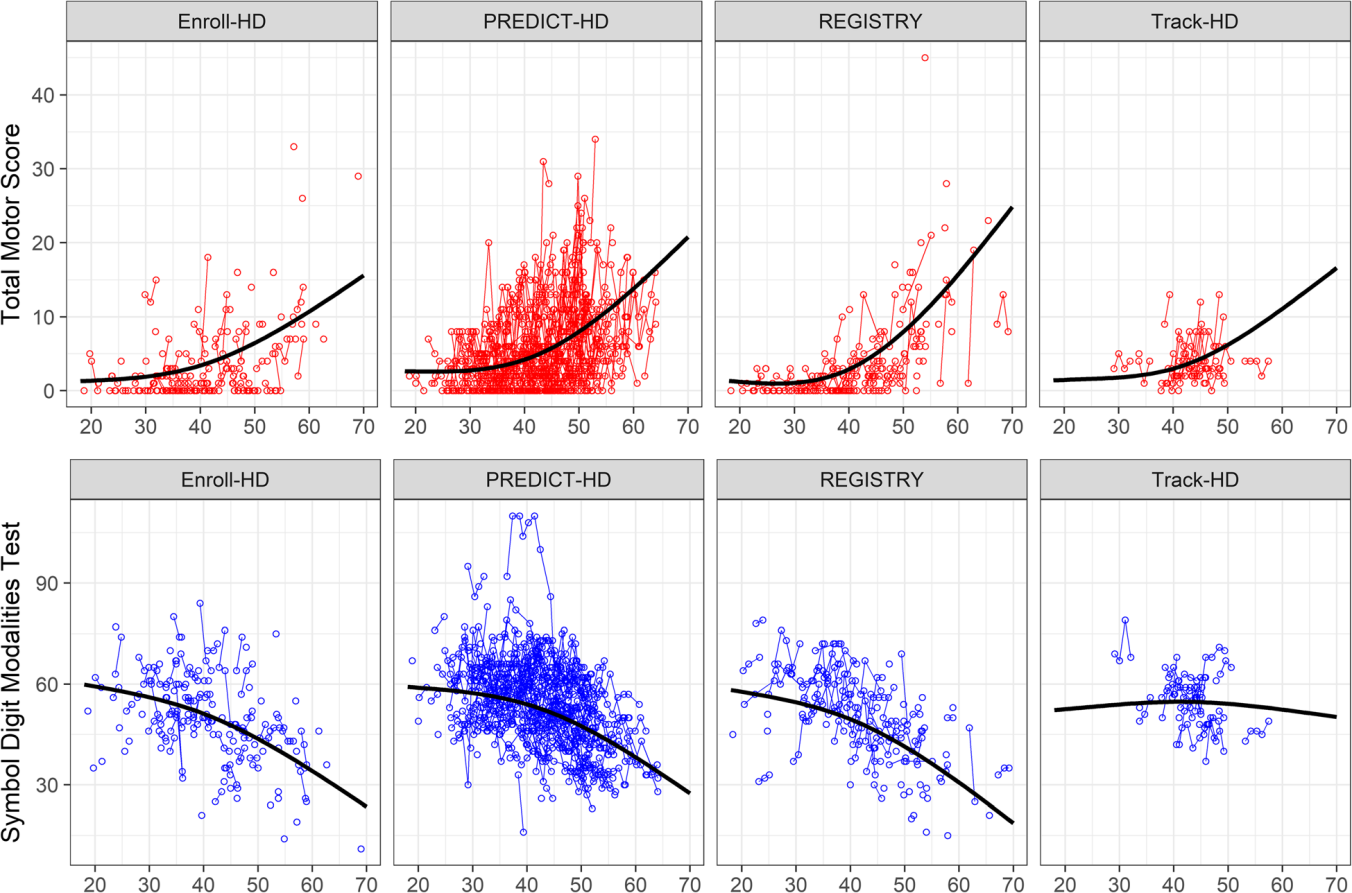
Age plots by study for participants with CAG = 42. Top row is observed total motor score (circles) by age with cubic spline curve (solid line), and the bottom row is the observed symbol digit modalities test with cubic spline curve

We sought to translate Eq. 1 to a JM. The first step in this translation was specifying the longitudinal model for the covariates. The LMM for each covariate was guided by previous work in HD. It is known that when tracking the TMS and SDMT prior to motor diagnosis, they each tend to display a non-linear but monotonic trajectory, with their levels being steady through younger ages and accelerating as the time of motor diagnosis approaches. For this reason, splines with few knots (i.e., one) have been suggested for modeling the longitudinal trajectories of these variables [26]. An example of observed data and fitted non-linear trajectories are illustrated in Figure 1. Age plots are paneled by study for participants with CAG = 42, which is the most frequently occurring expansion in every study. The top row shows observed TMS scores (circles) by age with an individual’s repeated measures connected by a thin line, and the cubic spline fit is shown by a thick line (the spline had a single knot at the median of the age distribution). The bottom row shows similar plots for SDMT.

In HD research it is important to model age interactions with CAG expansion, as the timing of motor diagnosis is negatively related to CAG expansion. Non-linear trends can occur at different ages for patients with different CAG expansions [13]. Thus, age and CAG expansion were disentangled from the CAP score in building the LMM.

For the LMM, suppose that *y*_*ij*,*k*_ denotes the outcome of the *k*^*th*^ variable (*k* = 1, 2) for the *i*^*th*^ patient (*i* = 1, …, *N*_*k*_) at the *j*^*th*^ time (*j* = 1, …, *n*_*i*,*k*_). To align the LMM notation with the survival notation, an outcome score can be denoted as *y*_*i*,*k*_(*t*_*ij*,*k*_). More abstractly, let *y*_*i*,*k*_(*t*) denote the outcome of a given patient at time *t* for a given variable (TMS or SDMT). The LMM is then,

2 $$ {\displaystyle \begin{array}{rr}{y}_{i,k}(t)=& \left({\beta}_{0,k}+{b}_{0i,k}\right)+\left({\beta}_{1,k}+{b}_{1i,k}\right){f}_1\left({\mathtt{AGE}}_i(t)\right)+\left({\beta}_{2,k}+{b}_{2i,k}\right){f}_2\left({\mathtt{AGE}}_i(t)\right)\\ {}+& {\beta}_{3,k}{\mathtt{CAG}}_i+{\beta}_{4,k}{\mathtt{CAG}}_i{f}_1\left({\mathtt{AGE}}_i(t)\right)+{\beta}_{5,k}{\mathtt{CAG}}_i{f}_2\left({\mathtt{AGE}}_i(t)\right)+{\epsilon}_{i,k}(t),\kern2.00em \end{array}} $$

where *f*_1_(·) and *f*_2_(·) are the piece-wise polynomials of the natural cubic spline with a single knot at the median of the age distribution; *β*_*l*,*k*_ is a fixed effect; *b*_*li*,*k*_ is a random effect; and *ϵ*_*i*,*k*_(*t*) is random measurement error. The random effects of the two outcomes, *b*_*li*,1_, *b*_*li*,2_ are assumed to have a joint-normal distribution with zero-means. It is further assumed that the random effects are uncorrelated with the random error.

A key to the JM interpretation is the partitioning *y*_*i*,*k*_(*t*) = *m*_*i*,*k*_(*t*) + *ϵ*_*i*,*k*_(*t*), where *m*_*i*,*k*_(*t*) is the true or underlying value of the *k*^*th*^ covariate at time *t*. We focus on the true value function, but there are additional functions of the longitudinal process that could be specified based on the research question of interest. For example, the instantaneous rate of change of the variables at time *t* could be the summary, or the cumulative process history of the covariates (i.e., the integral up to time *t*), or combinations of these functions [6].

Having specified the LMM, we now consider the survival submodel of the JM. As mentioned, CAG expansion has a well-established negative correlation with age of motor diagnosis [27]. Therefore, CAG expansion was specified as a time-invariant predictor in the survival submodel (CAG expansion was treated as fixed at birth). In addition to CAG expansion, the true value of each covariate (TMS and SDMT) at time *t* was included in the survival submodel. Assume that the hazard of motor diagnosis is conditional on CAG expansion and the true longitudinal processes of TMS and SDMT up to *t*. With this in mind, the survival submodel can be written as,

3 $$ {h}_i(t)={h}_0(t)\mathit{\exp}\left\{{\gamma}_1{\mathtt{CAG}}_i+{\alpha}_1{m}_{1i}^{\left(\mathtt{TMS}\right)}(t)+{\alpha}_2{m}_{2i}^{\left(\mathtt{SDMT}\right)}(t)\right\},\kern3.00em $$

where ${m}_{1i}^{\left(\mathtt{TMS}\right)}(t)$ is the true (unobserved) value of TMS, and ${m}_{2i}^{\left(\mathtt{SDMT}\right)}(t)$ is the true value of SDMT.

The predictors in Eq. 3 are correlated through the shared random effects in the longitudinal submodel (CAG expansion is also in the longitudinal submodel). The regression coefficients have the same interpretation as the log hazard ratios in the traditional proportional hazards model, with *γ*_1_ indexing the association of CAG expansion with the log hazard adjusting for the other predictors, *α*_1_ indexing the association of the TMS true value and the log hazard adjusting for the other predictors, and *α*_2_ indexing the association between the SDMT true value and the log hazard adjusting for the other predictors. Our focus will be on the survival submodel of Eq. 3, though there are applications in which the longitudinal submodel of Eq. 2 might be of prime interest.

**Fig. 2:**
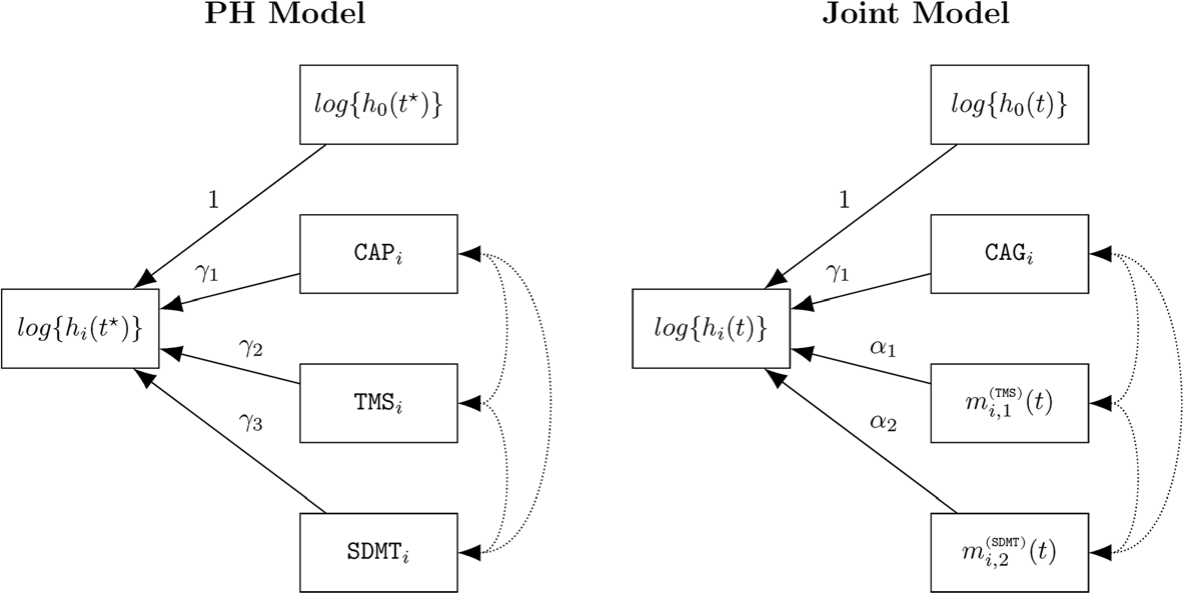
Diagram for the proportional hazards model (left) and the joint model survival submodel (right). The down-stream variable is the log hazard, which is a weighted combination of the up-stream variables, with the weights being the arrow labels. The dotted lines indicate correlation among the covariates; t* is time on study (t* = 0 is study entry), and *t* is age (*t* = 0 is birth)

Figure 2 shows diagrams for the proportional hazards model (left) and the JM survival submodel (right). In both diagrams, the down-stream log hazard is depicted as the sum of the log baseline hazard and the covariates, weighted by the regression coefficients labeled on the arrows. The dotted lines denote correlations among the covariates. As the figure indicates, both models have the same number of covariates, but the JM submodel exchanges CAG for CAP (both measured at study entry), and the longitudinal information of TMS and SDMT is used rather than the study-entry values of the covariates. The time metric of the proportional hazards model is time on study, denoted as *t*^⋆^, whereas the time metric the JM survival submodel is age, denoted as *t*.

### Estimation and inference

For the datasets of the analysis, none of the participants were observed from the time origin of birth. Rather, participants came under observation at their age of entry into their respective studies. Thus, there was a delayed entry into the risk set (delayed relative to birth), which is also known as left-truncation [28]. Let *T*_0*i*_ represent the age at which an individual comes into the study, with *T*_0*i*_ < *T*_*i*_. We assume the entry age of *T*_0*i*_ is not informative regarding HD progression.

In order to have participants enter the risk set at their proper times, a counting process approach was adopted for the survival submodel [1]. We coded the interval between the observed ages for two consecutive visits in a participant’s longitudinal series according to whether motor diagnosis occurred at the end of the interval or not (1 = motor diagnosis, 0 otherwise).

Estimation and inference for the JM rests on conditional independence assumptions [5]. For our analysis, the submodels of Eqs. 2 and 3 constitute the JM, along with the additional variance-covariance parameters of the random effects. It is assumed that conditional on the random effects, the submodels are independent (i.e., the event times and longitudinal outcomes), as are the repeated measures of the longitudinal submodel, and the longitudinal outcomes of TMS and SDMT. In addition to the independence assumptions, we further assume that given the observed history, the right-censoring mechanism and the mechanism that generates the longitudinal visits are independent of the true event times and future longitudinal measurements [5].

Maximizing the log-likelihood function of the JM can be accomplished using standard (i.e., frequentist) algorithms, with software such as the $\mathtt{joinRML}$ package [29]. An alternative adopted for our analysis is a Bayesian approach using the $\mathtt{JMbayes}$ package [30]. $\mathtt{JMbayes}$ offers flexibility in that it can accommodate non-continuous longitudinal covariates (e.g., a binary outcome) and diverse types of association structures for the longitudinal covariates (e.g., rate of change can be used as a predictor in the survival submodel). But the main motivation for adopting $\mathtt{JMbayes}$ is the extensive methods available for deriving individual-specific predictions.

In Bayesian analysis, the priors and the data likelihood are combined to yield posterior distributions for the parameters. These posterior distributions are the basis of all inference. For our JM, the posterior probability density is comparable to

4 $$ p\left(\theta, b\right)\propto \frac{\prod_{i=1}^N{\prod}_{k=1}^{K=2}{\prod}_{j=1}^{n_{i,k}}p\left({y}_{ij,k}|{b}_{i,k},\theta \right)p\left({T}_i,{\delta}_i|{b}_{i,k},\theta \right)p\left({b}_{i,k}|\theta \right)p\left(\theta \right)}{S\left({T}_{0i}|\theta \right)},\kern2.00em $$

where *θ* is the combined longitudinal, survival, and random effects parameters vector, and *b* is the random effects vector. The denominator is the adjustment for left-truncation consisting of the joint density of the delayed-entry survival function and the random effects (for details, see [31, 32]).

Our preoccupation is with the probability density relating to survival, which under the proportional hazards model is,

5 $$ {\displaystyle \begin{array}{rr}p\left({T}_i,{\delta}_i|{b}_{i,k},\theta \right)=& {\left[{h}_0\left({T}_i\right)\exp \left\{{\gamma}_1{\mathtt{CAG}}_i+{\alpha}_1{m}_{1i}^{\left(\mathtt{TMS}\right)}\left({T}_i\right)+{\alpha}_2{m}_{2i}^{\left(\mathtt{SDMT}\right)}\left({T}_i\right)\right\}\right]}^{\delta_i}\times \\ {}& \exp \left[-{\int}_0^{T_i}{h}_0(s)\exp \left\{{\gamma}_1{\mathtt{CAG}}_i+{\alpha}_1{m}_{1i}^{\left(\mathtt{TMS}\right)}(s)+{\alpha}_2{m}_{2i}^{\left(\mathtt{SDMT}\right)}(s)\right\} ds\right],\kern2.00em \end{array}} $$

with the second expression being the survival function.

The integral in Eq. 5 does not have a closed-form solution and numerical methods are needed for its evaluation. For the analysis we used the $\mathtt{JMbayes}$ Markov Chain Monte Carlo (MCMC) algorithm to sample from the posterior conditional distributions. We adopted standard diffuse priors, and the baseline hazard was estimated using a B-spline approach [30]. To facilitate convergence to the target posterior distributions, the MCMC procedure used an initial burn-in of 600 iterations. After burn-in, every 300th simulated value was retained until 1000 values were accumulated. For each posterior distribution we report the mean of the 1000 values as the parameter estimate, the SD as the measure of parameter variability (uncertainty), and the 2.5 and 97.5 quantiles as the 95% credible interval (CI). Graphical MCMC diagnostics (results not presented) included trace plots that showed reasonable consistency with steady states, lagged autocorrelation plots with very small values, and posterior density plots that were reasonably symmetric.

Each dataset was analyzed separately and the combined data was also analyzed. The combined study JM included a main effect for study in the survival submodel. This allowed for a study-specific effect with the baseline hazard being arbitrarily defined for Enroll-HD.

CAG expansion was limited to the range of 40 to 48 because 40 is the boundary of full penetrance and the data were sparse above 48. Individual variables had very little missing data among the studies, and complete cases were used for the analysis. The value of 42 was subtracted from CAG expansion (the mode among the studies) in order to avoid large discrepancies in the regression coefficients.

### Individual-specific predictions

Once the posterior of the JM was estimated, predicted survival probabilities for each individual were computed. For our data, each participant had pre-diagnosis (pre-risk) longitudinal data measured from their age of study entry up to (but not including) their age of diagnosis or censoring. The last pre-diagnosis age was associated with a survival probability of 1 because it was the last known age of no risk for diagnosis (if a participant was censored, then the last value was the censoring age). Suppose the last known age of no risk for diagnosis is denoted as *t*. We were interested in the probability of survival beyond an age older than *t*, say *u* (conditional on the data and the covariates). The notation *π*_*i*_(*u*| *t*) is used to indicate the *i*^*th*^ person’s probability of diagnosis being greater than or equal to age *u*, given they were not diagnosed up to age *t*.

An estimate of *π*_*i*_(*u*| *t*) was computed by resampling from the posterior distributions produced from the MCMC procedure discussed above using the $\mathtt{survfitJM}\left(\right)$ function. For each resampling iteration, there was a single random draw from each relevant posterior distribution, including the random effects, and then the individual-specific survival probability was computed (for details see [5, 32]). The process was repeated 500 times and the mean survival probability among the resamplings for a given age was taken as the predicted individual-specific survival probability. The standard error was the SD among the resamplings, which was then used to compute the 95% CI for the survival curves.

There were two predicted scores of interest: (1) age at which the model predicted diagnosis, and (2) a residual indicating the discrepancy between an individual’s observed diagnosis status at time of diagnosis or censoring and their model-predicted status. Both types of predicted scores were based on the individual-specific predicted cumulative hazard.

**Fig. 3:**
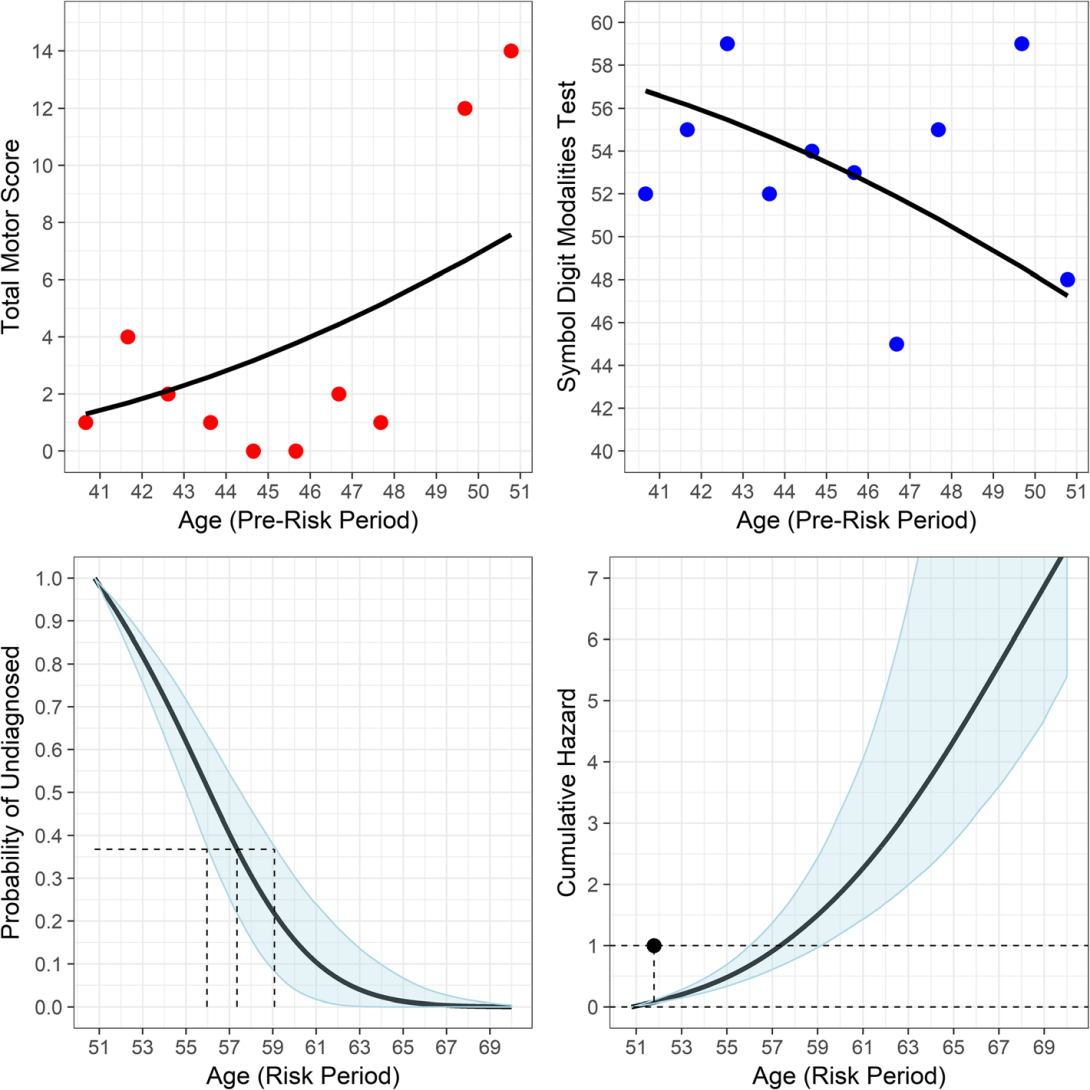
Joint modeling results for one participant of the analysis. Upper panels show observed longitudinal variable scores (points) and model-based predictions (lines). Lower panels show predicted survival curve with credible interval (left) and predicted cumulative hazard (right)

Once the survival probabilities were obtained by the resampling method, the predicted cumulative hazard for an individual was computed assuming a unit exponential failure process. Suppose ${\hat{\varLambda}}_i\left(u|t\right)$ is the predicted cumulative hazard up to time *u* for the *i*^*th*^ participant (assuming they were undiagnosed up to *t*). Then the cumulative hazard was computed as ${\hat{\varLambda}}_i\left(u|t\right)=-\mathit{\log}\left({\hat{\pi}}_i\left(u|t\right)\right)$. Individual-specific predictions for one of the participants are illustrated in Figure 3. The top two panels show TMS (left) and SDMT (right) observed scores (filled points) and the individual-specific predicted curves (see below). The bottom panel shows the predicted survival probabilities (left) and the cumulative hazard (right) along with the 95% CI. The top panels span the pre-diagnosis age range of *t* ∈ [40.51,50.78] for the participant in which TMS and SDMT were observed and there was no risk of diagnosis (looking retrospectively). Conversely, the bottom panels span the age range of *t* > 50.78, in which there was a risk for diagnosis (the participant in question was diagnosed in the risk period).

Under a counting process approach to survival analysis [1], ${\hat{\varLambda}}_i\left(u|t\right)$ represents the number of diagnoses that occur up to time *u*, assuming that diagnoses can be accumulated for an individual. Less formally, we refer to ${\hat{\varLambda}}_i\left(u|t\right)$ as the accumulated risk up to age *u*. Then, ${\hat{\varLambda}}_i\left(u|t\right)=1$ represents the model-based diagnosis event, ${\hat{\varLambda}}_i\left(u|t\right)<1$ represents accumulated risk that has not yet reached the threshold of diagnosis, and ${\hat{\varLambda}}_i\left(u|t\right)>1$ represents “excess” risk that accumulates after diagnosis.

Age at the model-predicted diagnosis was taken to be the age associated with ${\hat{\varLambda}}_i\left(u|t\right)=1$, or equivalently, the age associated with $\hat{\pi}\left(u|t\right)=\mathit{\exp}\left(-1\right)=.3679$. A grid search was conducted for each individual to find their predicted age. The lower right panel of Figure 3 shows that the cumulative hazard reached the diagnosis threshold of 1 for the participant at age 57.35. The lower left panel shows the same in terms of survival probability, with the middle vertical dashed line indicating the age associated with the model-predicted diagnosis (with threshold of ${\hat{\pi}}_i\left(u|t\right)=.3679$), and the outer dashed lines indicating the CI bounds.

A martingale-like residual [33] was computed for the time at diagnosis or censoring, *T*_*i*_. The residual is defined as *r*_*i*_(*T*_*i*_| *t*) = *δ*_*i*_ − *Λ*_*i*_(*T*_*i*_| *t*), where *δ*_*i*_ is the diagnosis indicator mentioned previously. The residual range is (−∞, +1], and the residual indicates the agreement between the observed diagnosis status and the status predicted by the model. Unlike the traditional martingale residual, the *r*_*i*_(*T*_*i*_| *t*) do not sum to 0 in general due to their individual-specific nature. The residual is always a negative value for a censored participant, but the more negative the value the greater the discrepancy between the observed status and the model-predicted status (in the form of accumulated risk). Diagnosed participants can have negative or positive values. The bottom right panel of Figure 3 shows the observed diagnosis status of *δ*_*i*_ = 1 at *T*_*i*_ = 51.78 with a filled circle. The residual is the vertical distance between the filled circle and the cumulative hazard, which is denoted by a vertical dashed line.

To help induce the residual distribution to be more symmetric, the transformation for the deviance residual was used [33],

$$ {d}_i\left({T}_i|t\right)=\mathit{\operatorname{sign}}\left[{r}_i\left({T}_i|t\right)\right]\times \sqrt{-2\left[{r}_i\left({T}_i|t\right)+{\delta}_i\mathit{\log}\left({\delta}_i-{r}_i\left({T}_i|t\right)\right)\right]}, $$

where *sign*(·) takes the value of + 1 if the martingale residual is positive and − 1 otherwise.

We close this section by noting that individual-specific predictions can also be made for the longitudinal covariates. For our analysis the method was to use the mean posterior fixed effects and the mean posterior random effects from the LMM submodel. We highlight that the MCMC algorithm generates a multivariate posterior random effects distribution for each participant, so that the means of the posterior random effects are specific to an individual (though the fixed effects are not). The posterior means were used with the observed design matrices for the fixed effects and random effects to compute predicted values. For example, based on the LMM submodel in Equation 2, the predicted TMS values (*k* = 1) for the *i*^*th*^ participant were computed as

$$ {\hat{y}}_{i,1}(t)=\left({\hat{\beta}}_{0,1}+{\hat{b}}_{0i,1}\right)+\left({\hat{\beta}}_{1,1}+{\hat{b}}_{1i,1}\right){f}_1\left({\mathtt{AGE}}_i(t)\right)+\dots +{\hat{\beta}}_{5,1}{\mathtt{CAG}}_i{f}_2\left({\mathtt{AGE}}_i(t)\right). $$

The smooth curves in the top panels of Figure 3 show the predicted longitudinal covariate values for one participant in the analysis.

### External validation

One indication of the usefulness of a model developed in a single sample is the extent to which the model is transportable to other data, or the extent to which we can validly apply the model to external data [34]. Assessment of external validity for the JM focused on how well the model estimated in one study (the training dataset) was able to discriminate among diagnosed and pre-diagnosed participants in the other studies (the test datasets). Discrimination was estimated using a time-dependent AUC statistic [35] computed with the function $\mathtt{aucJM}\left(\right)$[30].

The most common AUC measure in proportional hazards survival analysis is Harrell’s *C* [36], which is the probability that a participant who is diagnosed at an older age also has a higher predicted survival probability than a second participant who is diagnosed at a younger age. For the proportional hazards model there is one survival curve for a subgroup with a particular combination of covariates (e.g., males with CAG = 42). Two people of the subgroup with different ages of diagnosis will have different survival probabilities, with the older diagnosed having the higher survival probability (lower probability of diagnosis). This strict ordering makes Harrell’s *C* relatively straight-forward to compute and interpret in traditional survival analysis [37].

Strict ordering does not hold under the JM scenario because the survival curves are individual-specific (the subgroup is generally of size 1). The start age and slope of an individual’s survival curve depend on the vector of longitudinal TMS and SDMT observations, as well as the CAG expansion. The result is a staggering of individual survival curves with various start ages and rates of change. In this situation the survival curves of two participants can cross, meaning the ordering based on survival probabilities can change depending on the window of evaluation, which can result in an ambiguous interpretation.

Time-dependent AUC addresses the above issue by aligning individuals to a common start age and compares individuals in reference to a fixed age window. Denote the start age of the window as *v* and the end age as *w*. Consider participants who have a longitudinal covariate history up to *v*. A comparison is defined for a pair of comparable participants, comparable here meaning that the first participant (*i*) converts to a motor diagnosis within (*v*, *w*], and the second participant *i*^′^ converts after *w*. The pair is concordant if the survival probability for participant *i* at *w* is less than the survival probability of participant *i*^′^. That is, concordance occurs when the model assigns a higher survival probability to the participant who did not convert within the age window. AUC is defined as the probability of concordance, and the AUC estimator of $\mathtt{aucJM}\left(\right)$ accounts for both concordance and censoring. The AUC statistic is computed as the sum of the proportion of concordant pairs among the total number of comparable pairs and the weighted proportion of pairs that cannot be compared due to censoring [30, 32].

Time-dependent AUC constrains who can be analyzed because individuals must have longitudinal data preceding *v*. In order to include a wide variety of participants, three windows were considered with start ages of *v* = 30,40,50. Because HD has a relatively slow progression, 5-year and 10-year windows were considered. For each window, the estimates of one study (based on the posterior predictive distributions) were used for discrimination in the remaining studies.

## Results

### Comparison of coefficients among studies

Estimated regression coefficients of the survival submodel are shown in Table 2, along with the posterior SDs (in parentheses) and the 95% CI bounds (in brackets). Results are shown for each study estimated in isolation, and also for the combined data (last row). It was of interest to examine whether a parameter could be 0 based on its posterior distribution. To this end, we evaluated if 0 was in the CI for each effect.

**Table 2:** Parameter estimates (SD)[95% CI] for the multivariate joint model survival submodel

	CAG Expansion	Total Motor Score	Symbol Digit
Enroll-HD	0.294(0.057)[0.188, 0.408]	0.041(0.011)[0.018, 0.063]	−0.005(0.008)[− 0.023, 0.012]
PREDICT-HD	0.342(0.050)[0.245, 0.436]	0.102(0.013)[0.077, 0.128]	−0.025(0.008)[− 0.040, − 0.011]
REGISTRY	0.354(0.071)[0.211, 0.491]	0.064(0.015)[0.034, 0.095]	−0.023(0.013)[− 0.048, 0.003]
Track-HD	0.572(0.128)[0.319, 0.822]	0.126(0.071)[−0.015, 0.274]	− 0.026(0.013)[− 0.054, − 0.000]
Combined	0.350(0.031)[0.293, 0.410]	0.065(0.008)[0.051, 0.080]	−0.016(0.005)[− 0.025, − 0.006]

Note. *CAG* cytosine-adenine-guanine expansion. Combined model added a study-specific main effect (see text)

The estimates for CAG expansion were positive among all the studies, indicating that larger lengths were associated with greater hazard of motor diagnosis. The CI did not contain 0 for any study, or for the combined data.

The estimates for TMS were also positive, and none of the CIs contained 0, except for Track-HD. The estimates for SDMT were all negative, which indicated that a lower value of SDMT (worse performance) was associated with greater hazard of motor diagnosis. The CIs for Enroll-HD and REGISTRY contained 0, but the CIs for the other two studies did not.

For the combined data, the sign of the coefficients were positive for CAG and TMS, and negative for SDMT. The CI for each effect did not contain 0.

### AUC external validation

The AUC results are shown in Table 3. Results for 5-year and 10-year age windows are shown for each study on which the model was trained (the other studies provided the test data). The number of individuals at-risk for the age window is also indicated (determined by the start age and the test data). The mean 5-year AUC = .83 (range .77–.90), and the mean 10-year AUC = .86 (range .82–.92). The table indicates that the AUC decreased as the start age increased, and the 5-year AUC was smaller than the 10-year for each start age. On average, the smallest AUCs were trained on Enroll-HD, and the largest were trained on Track-HD.

**Table 3:** External validity results showing the 5-year and 10-year area under the curve (AUC) by training study and start age

Training Study	Start Age	At-Risk	5-YearAUC	10-YearAUC
Enroll-HD	30	138	0.804	0.825
PREDICT-HD	30	72	0.842	0.861
REGISTRY	30	134	0.826	0.841
Track-HD	30	160	0.898	0.915
Enroll-HD	40	239	0.782	0.818
PREDICT-HD	40	113	0.822	0.865
REGISTRY	40	221	0.828	0.846
Track-HD	40	231	0.876	0.897
Enroll-HD	50	173	0.774	0.822
PREDICT-HD	50	58	0.827	0.872
REGISTRY	50	160	0.812	0.829
Track-HD	50	173	0.861	0.889

### Predicted scores

**Fig. 4:**
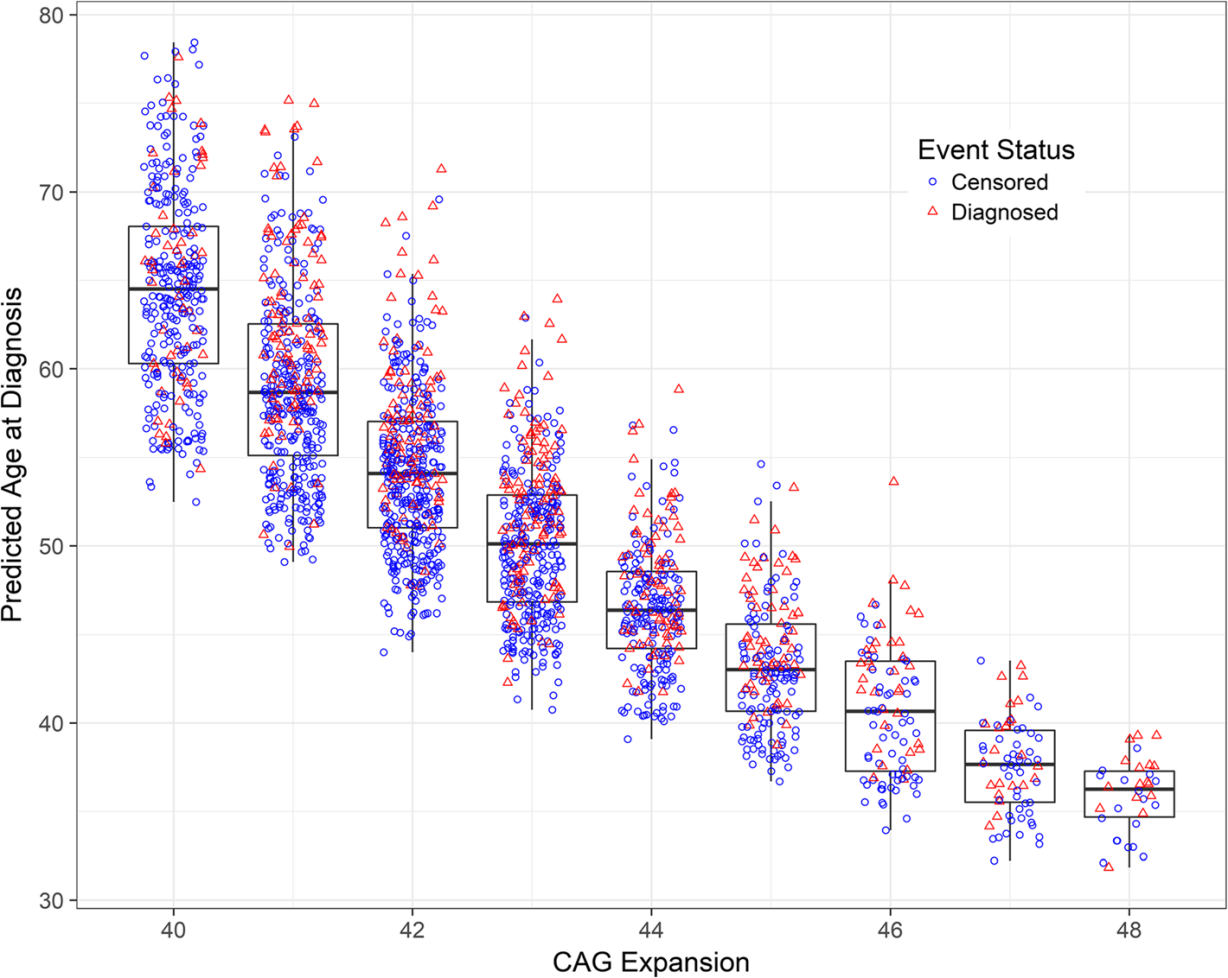
Predicted age at diagnosis (with boxplot) by CAG expansion and diagnosis status

The timing of motor diagnosis is of high interest in HD research. Motor diagnosis indicates a major progression event and it is important in determining eligibility for clinical trials. Figure 4 shows boxplots of predicted age of motor diagnosis as a function of CAG expansion and diagnosis status (circle for censored and triangle for diagnosis). As the figure shows, the median age of diagnosis decreased as CAG expansion increased, and there was substantial age variability. Figure 4 is similar to the pattern of results found by other researchers who analyzed only prospectively diagnosed individuals [27]. The novelty here is that we include both prospectively diagnosed and censored individuals.

Another type of predicted score with applicability to HD research is the deviance residual. Residuals are typically used to examine (in)consistency with statistical assumptions, but in the present context they have an alternative use for HD research. Since the discovery of the HD genetic mutation, there has been a search for additional genetic variants using genome-wide association studies (see e.g., [38]). It is common in such studies to examine phenotypic extremes, with the motivation being that those in the tails on either side of a distribution are most likely to provide an informative comparison [39]. The phenotypic extremes are often based on residuals from a prediction model that includes risk factors. In the current context, extreme deviance residuals index either deficient or excessive risk of motor diagnosis. Comparing genetic information among the extremes of the residual distribution might help account for variability in the timing of motor diagnosis.

**Fig. 5:**
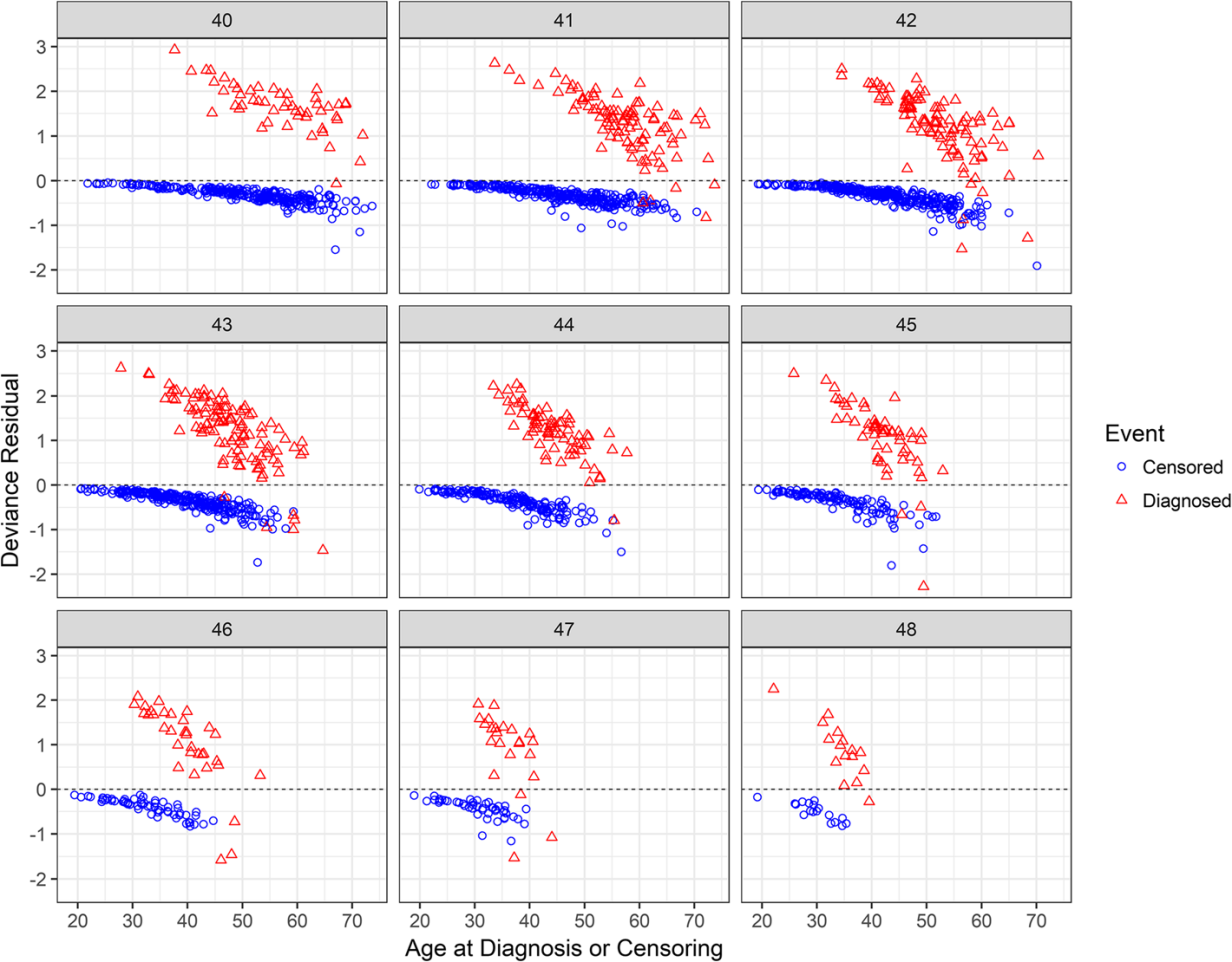
Deviance residual by age, CAG expansion, and event status

Figure 5 shows the deviance residual as a function of age, CAG expansion, and diagnosis status. The closer a residual is to 0, the greater the agreement between the observed event status (diagnosis or censoring) and the model-based risk. For the censored participants, the deviance residuals were very close to 0 for the younger ages, but became increasingly more negative with age, meaning older participants did not convert to a diagnosis even as their risk to do so increased. For the prospectively diagnosed participants, the deviance residuals were farthest from 0 in the positive value direction for the younger ages, but decreased towards 0 with age (resulting in some residuals being negative). Thus, the younger diagnosed participants converted even though their risk to do so was relatively low.

Based on the definition of the deviance residuals, certain individuals in Figure 5 might be classified as being diagnosed “early” or “late”. In each CAG panel, the youngest diagnosed participants at the upper left were diagnosed early, in the sense that they converted to a diagnosis with very low model-predicted risk. Conversely, the oldest censored participants at the lower right were late to be diagnosed because they had relatively high risk but did not convert to a diagnosis in the observed time period. We also note that the censored participants who were young tended to be “on time” for diagnosis in the sense that they had low model-predicted risk and did not covert to a diagnosis. The diagnosed participants who were relatively old tended to also be “on time”.

## Discussion

We considered a JM for the prediction of the hazard of HD motor diagnosis with two longitudinal clinical variables (TMS and SDMT) and one time-invariant genetic variable (CAG expansion). The JM was initially estimated separately on four studies, and then estimated on the combined data with an enhanced JM that had a study-specific effect. The results show that the external validity performance of the JM was relatively strong, in the respect that the time-dependent AUC values in the test data were high by traditional standards.

Reference values for external validity AUCs are provided by a recent survey in oncology and cardiovascular disease [40]. The survey found a mean AUC = 0.78 among studies, with 1st quartile AUC = 0.69 and 3rd quartile AUC = 0.88. Our results show that the mean time-dependent AUCs had values that were not much smaller than the 3rd quartile of the survey. The relatively high external values boost confidence that the JM considered in this study will have adequate discrimination performance in a new HD sample from the same population of pre-diagnosed patients

External validity performance was evaluated with the time-dependent AUC because discrimination among diagnosed and pre-diagnosed individuals is especially meaningful in HD research, and AUC reflects a metric familiar to clinical researchers [25]. An alternative approach is to evaluate predictive performance using a calibration measure that quantifies the agreement between observed outcomes and model-based predictions [41]. One example of a calibration measure is the Brier score, which in the survival context is defined as the expected squared discrepancy between the diagnosis status and the model-predicted survival probability [42]. In the JM context, a Brier-type measure for a time window has been proposed by Henderson et al. [43], which can be computed using the $\mathtt{prederrJM}\left(\right)$ function of $\mathtt{JMbayes}$[30].

A caveat regarding the external validity analysis is that there may have been some participant overlap among studies. Of the four studies analyzed, Enroll-HD is the most recent and the only one currently active. After termination of PREDICT-HD and Track-HD, a number of participants were known to have transitioned to Enroll-HD. Furthermore, there was a concerted effort to transition all REGISTRY participants to Enroll-HD [17]. Through the use of a common ID number, most of the participants who had transitioned were identified, and only the data from their initial study was used. However, it is possible that not all the participants that transitioned had an ID that allowed for their identification. A definitive analysis of overlap is not possible because necessary identifying information, such as birth dates, is not available for purposes of anonymity. We highlight that PREDICT-HD and Track-HD participants were known to be exclusive to their studies [21], and REGISTRY participants were transitioned over to Enroll-HD in a careful manner suggesting that all overlap could be successfully accounted for by the common ID. Thus, we believe that any remaining data overlap among the studies was inconsequential regarding the overall findings.

The relatively strong external validation performance of the JM considered in this study does not suggest the model is optimal. There could be alternative models with similar or better performance. The estimated regression coefficients of the survival submodel (Table 2) show that CAG expansion was the most important predictor, followed by TMS and SDMT. It is unclear if a JM having CAG expansion and only one or the other of the longitudinal covariates would perform similar to the multivariate JM considered here. Furthermore, CAG expansion had both an indirect effect and a direct effect on the hazard of motor diagnosis. The indirect effect resulted from including CAG expansion in the longitudinal submodels, whereas the direct effect resulted from including CAG expansion in the survival submodel. It might be of interest to evaluate whether both types of effects are required.

Future research might focus on several candidate models, and there are a number of measures that can be used for Bayesian model selection. We note that the AUC and Brier-like measures of the $\mathtt{JMbayes}$ package are Bayesian in nature because they use survival probabilities estimated from the appropriate predictive posterior distributions. In terms of model selection, AUC may not be a desirable index. In the context of proportional hazards modeling, AUC has been shown to be relatively insensitive to model differences, unless the effect sizes are very large [44, 45]. Brier-type measures tend to shown greater sensitivity and might be preferred for model selection [46]. Additional tools for Bayesian model selection include the deviance information criterion (DIC) [47], the conditional predictive ordinate [48], and the log pseudo-marginal likelihood (LPML) [49]. Recent extensions of the DIC and LPML allow for separate model selection among the survival and longitudinal submodels [50].

Our study illustrates types of predicted scores that might be useful for individual-specific disease characterization. The predicted scores consisted of predicted age of HD motor diagnosis and a deviance-type residual indicating the extent of agreement between observed and model-based diagnosis status. Predicted age at diagnosis can be used to help characterize an individual’s disease state. The number of years from a person’s current age to their predicted age of diagnosis offers an indication of the extent of progression, with a small difference representing relatively advanced progression and a large difference representing the converse. Such indexing might be important for timing the administration of interventions or identifying appropriate participants for clinical trials. To date, most HD clinical trials have targeted the period shortly after diagnosis [51]. However, new treatments are being developed to target the period shortly before diagnosis. The difference between current age and predicted age of onset can be used to identify individuals who might be appropriate for clinical trials of such treatments.

Previous work has focused on observed age of motor diagnosis only for those who prospectively convert to a diagnosis [13, 27]. A potential advantage of the JM approach is that predicted age of motor diagnosis can be computed for both censored and diagnosed participants. Thus, all the gene-expanded individuals of a study can be characterized in terms of their predicted progression, whether they have reached motor diagnosis or not. Despite a majority of censoring in the studies considered here, the plot of predicted age of diagnosis by CAG expansion (Figure 4) is very similar to plots using only diagnosed individuals [13, 27].

One use for the deviance residual is to serve as a phenotype in a future genetic analysis. In the time since the HD gene mutation discovery, there has been a continued search for additional genetic modifiers of HD [38, 52]. A common approach in genetic modifier discovery studies is to compute a residual based on observed status and a model-predicted risk score [53]. After computing a residual for each person, all individuals are ranked, and the upper and lower extremes are selected for analysis (say, the upper/lower 20%). Use of the extremes is an enrichment strategy that tends to improve power to discover genetic modifiers and detect their association with a phenotype [54]. The deviance-like residual can be used in such a manner to potentially identify genetic modifiers of the timing of diagnosis.

A complication of moving from a traditional proportional hazards model to a JM is that predicted scores are not simple to produce. In the case of the traditional proportional hazards model, it is typical to use the estimated linear predictor as a risk score formula [55] (see the diagram at left in Figure 2). In fact, such a risk score formula for HD motor diagnosis has been developed [21]. The advantage of the linear predictor risk score is that it is easily computed, given that a new or existing participant has measured values for the variables in the equation. In contrast, predicted scores of the JM cannot be computed analytically, but rather require computer simulation and a fitted model object. An additional complication is that the MCMC method discussed above is relatively time-intensive. The JM for the combined data that served as the basis for the predicted scores took approximately 3 h to run on a PC laptop with an Intel Core i7 processor.

Despite the added complexity, predicted values from the JM are preferable because they are likely to be more precise for an individual. Predictions from the proportional hazards model apply at the group level to those who share common values of the study-entry covariates. It is not surprising that such predictions can be quite inaccurate at the individual level [56]. In contrast, longitudinal covariate information and random effects are considered in the JM, which are unique for each individual. The result is greater individual-level prediction accuracy [6]. Thus, the complexity of computing predicted scores with JM is thought to be worth the gain in precision.

Joint modeling has previously been used in HD research [13, 57]. The novelty of this study is that we considered multiple longitudinal covariates, examined external validity performance, and proposed novel individual-specific predictions. Another difference is that we used age as the time metric (with origin at birth), rather than time on study (with origin at study entry). In the traditional survival setting, predictions from a model that uses time on study can be equivalent or approximately so to a model that uses age, provided the linear predictor is complex enough (e.g., includes non-linear terms) [58]. There is no such equivalence in the JM context due to the greater complexity introduced by the random effects. Changing the time metric in the longitudinal submodel will change the variance components of the random effects, which can result in quite different individual-level predictions. Therefore, attention needs to be given to the selection of the time metric prior to the analysis. We agree with the argument made by other researchers that age is the natural metric for longitudinal observational studies [59–61], including the HD studies considered here. Given the non-equivalence of JM results under a change of time metric, we recommend that age be used with adjustment for delayed entry.

## Conclusions

Joint models are an improvement over traditional survival models because they consider all the longitudinal observations of covariates that are predictive of the event of interest. Predictions from joint models have greater accuracy because they are tailored to account for individual variability. These predictions can provide relatively accurate characterizations of individual disease progression, which might be important for the timing of interventions, qualification for appropriate clinical trials, and additional genotypic analysis. This study illustrates the usefulness of JM for analyzing the HD datasets, but the approach is applicable to a wide variety of diseases.

## Abbreviations

HD: Huntington’s disease
JM: Joint modeling
SDMT: symbol digit modalities test
TMS: total motor score
UHDRS: Unified Huntington’s Disease Rating Scale
